# Supplementary figures and images for: Delimiting the boundaries of sesamoid identities under the network theory framework
Source: PeerJ. 2020 Aug 17;8:e9691. doi: 10.7717/peerj.9691 (PMC7439958; doi:10.7717/peerj.9691)

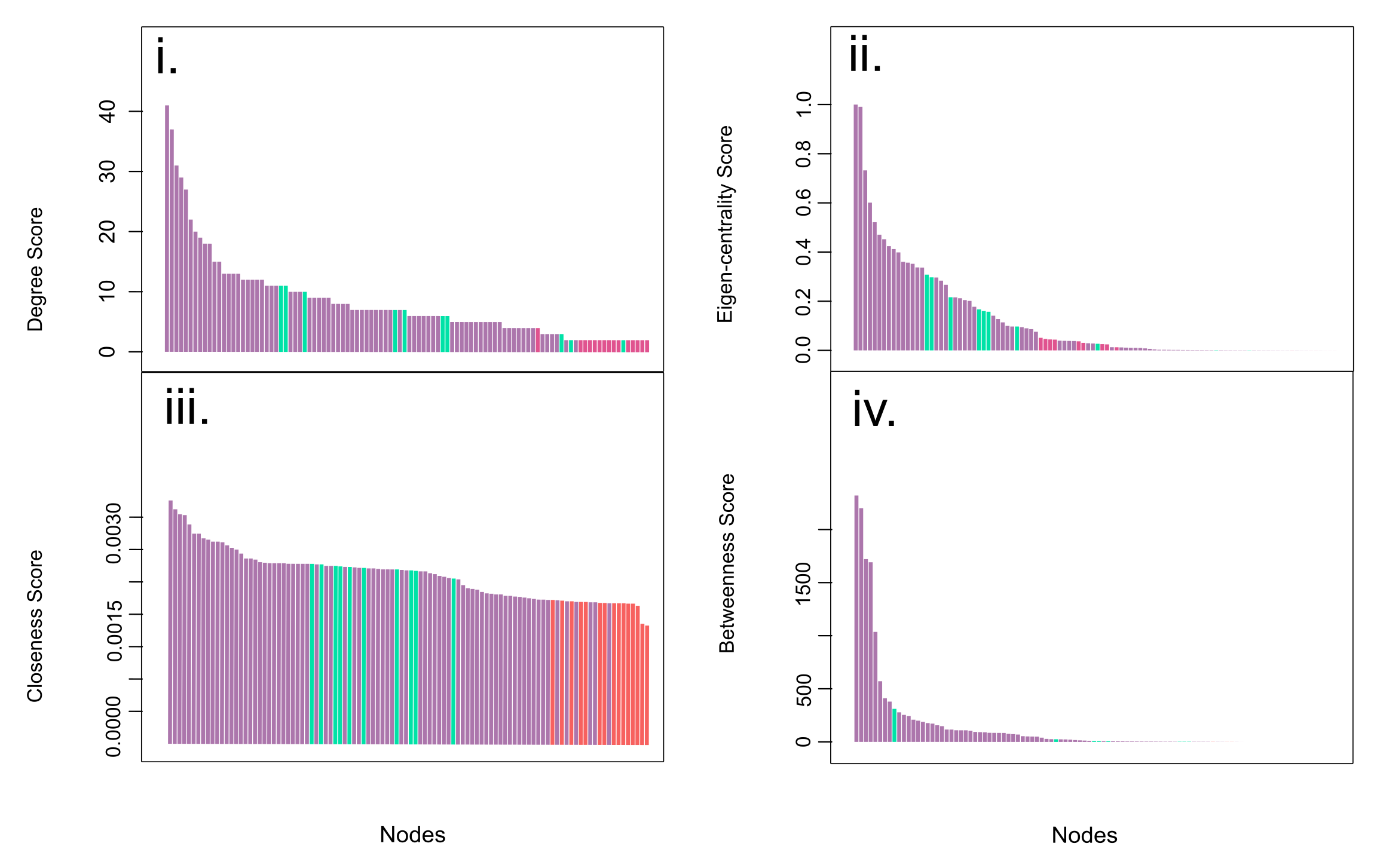

Supplement: Supplemental Information 1 — Centrality indicators bar plots. Each plot (i-iv) contains a bar plot of Degree, Eigen-centrality, Closeness and Betweenness values of every node of the network. Bar are ordered from the higher (left) to the lower values (right). Node categories are represented by colors. Non-sesamoid in purple, embedded sesamoids in green, and glide sesamoids in red. [file peerj-08-9691-s001.png]
