# Supplementary material for: Delimiting the boundaries of sesamoid identities under the network theory framework
Source: PeerJ. 2020 Aug 17;8:e9691. doi: 10.7717/peerj.9691 (PMC7439958; doi:10.7717/peerj.9691)
Supplement: Supplemental Information 3 — Two first columns indicate pairs of connected elements. Third column indicates the connection type. Abbreviations: F: finger FP: forelimb phalanx, HF: hindlimb phalanx, (f) forelimb, (h): hindlimb, T: toes, V: vertebra. [file peerj-08-9691-s003.pdf]

Supplemental Table S1: Adjacency matrix.

Legend: Two first columns indicate pairs of connected elements. Third column indicates the connection type. Abbreviations: F: finger FP: forelimb phalanx, HF: hindlimb phalanx, (f) forelimb, (h): hindlimb, T: toes, V: vertebra.

First element	Second element	Type of connection
cranium	atlas	Joint
atlas	V2	Joint
V2	V3	Joint
V3	V4	Joint
V4	V5	Joint
V5	V6	Joint
V6	V7	Joint
V8	sacral vertebra	Joint
sacral vertebra	urostyle	Joint
sacral vertebra	ilium	Joint
sacral vertebra	sacral sesamoid	ligament
sacral sesamoid	ilium	ligament
ilium	ischium	Joint
ilium	pubis	Joint
ischium	pubis	Joint
suprascapula	cleithrum	Joint
cleithrum	scapula	Joint
scapula	clavicle	Joint
clavicle	coracoid	Joint
clavicle	episternum	Joint
episternum	omosternum	Joint
clavicle	procoracoid	Joint
clavicle	epicoracoid	Joint
procoracoid	epicoracoid	Joint
coracoid	epicoracoid	Joint
coracoid	mesosternum	Joint

epicoracoid	mesosternum	Joint
epicoracoid	episternum	Joint
mesosternum	xiphisternum	Joint
clavicle	humerus	Joint
coracoid	humerus	Joint
scapula	humerus	Joint
humerus	radioulna	Joint
radioulna	radiale	Joint
radioulna	ulnare	Joint
radiale	ulnare	Joint
radiale	EL Y(F)	Joint
EL Y(F)	PREP PROX	Joint
PREP PROX	PREP DIST	Joint
radiale	CARPAL 2	Joint
radiale	CARPAL 3-4-5	Joint
ulnare	CARPAL 3-4-5	Joint
radiale	METC 2	Joint
CARPAL 2	METC 2	Joint
EL Y(F)	METC 2	Joint
CARPAL 3-4-5	METC 3	Joint
CARPAL 3-4-5	METC 4	Joint
CARPAL 3-4-5	METC 5	Joint
METC 2	FFI D2	Joint
FFI D2	FFII D2	Joint
METC 3	FFI D3	Joint
FFI D3	FFII D3	Joint
METC 4	FFI D4	Joint
FFI D4	FFII D4	Joint
FFII D4	FFIII D4	Joint
METC 5	FFI D5	Joint
FFI D5	FFII D5	Joint

FFII D5	FFIII D5	Joint
HFII D1	HFI D1	Joint
HFI D1	METT 1	Joint
HFII D2	HFI D2	Joint
HFI D2	METT 2	Joint
HFIII D3	HFII D3	Joint
HFII D3	HFI D3	Joint
HFI D3	METT 3	Joint
HFIV D4	HFIII D4	Joint
HFIII D4	HFII D4	Joint
HFII D4	HFI D4	Joint
HFI D4	METT 4	Joint
HFIII D5	HFII D5	Joint
HFII D5	HFI D5	Joint
HFI D5	METT 5	Joint
METT 1	METT 2	Joint
METT 2	METT 3	Joint
METT 3	METT 4	Joint
METT 4	METT 5	Joint
PREH DIST	PREH PROX	Joint
EL Y(H)	METT 1	Joint
EL Y(H)	PREH PROX	Joint
PREH PROX	METT 1	Joint
EL Y(H)	TARSAL 1	Joint
TARSAL 1	METT 2	Joint
TARSAL 1	TARSAL 2-3	Joint
TARSAL 2-3	METT 2	Joint
TARSAL 2-3	METT 3	Joint
tibiale	EL Y(H)	Joint
tibiale	TARSAL 1	Joint
tibiale	TARSAL 2-3	Joint

fibulare	TARSAL 2-3	Joint
fibulare	METT 5	Joint
tibiale	TIBFIB	Joint
fibulare	TIBFIB	Joint
TIBFIB	femur	Joint
TIBFIB	femur	Tendo-muscle
HFI D1	glide mett D1	Tendo-muscle
glide mett D1	METT 1	Tendo-muscle
HFI D2	glide mett D2	Tendo-muscle
glide mett D2	METT 2	Tendo-muscle
HFII D3	glide inter HFII-I D3	Tendo-muscle
glide inter HFII-I D3	HFI D3	Tendo-muscle
HFI D3	glide mett D3	Tendo-muscle
glide mett D3	METT 3	Tendo-muscle
HFIII D4	glide inter HFIII-II D4	Tendo-muscle
glide inter HFIII-II D4	HFII D4	Tendo-muscle
HFII D4	glide inter HFII-I D4	Tendo-muscle
glide inter HFII-I D4	HFI D4	Tendo-muscle
HFI D4	glide mett D4	Tendo-muscle
glide mett D4	METT 4	Tendo-muscle
HFII D5	glide inter HFII-I D5	Tendo-muscle
glide inter HFII-I D5	HFI D5	Tendo-muscle
HFI D5	glide mett D5	Tendo-muscle
glide mett D5	METT 5	Tendo-muscle
TIBFIB	CARTILAGO SESAMOIDE	tendo-muscle
CARTILAGO SESAMOIDE	Ses1 aponeurosis	tendo-muscle
CARTILAGO SESAMOIDE	Ses2 aponeurosis	tendo-muscle
CARTILAGO SESAMOIDE	HFI D1	tendo-muscle
CARTILAGO SESAMOIDE	HFI D2	tendo-muscle

CARTILAGO SESAMOIDE	HFI D3	tendo-muscle
femur	OS SESAMOIDES	tendo-muscle
TIBFIB	OS SESAMOIDES	tendo-muscle
OS SESAMOIDES	Ses1 aponeurosis	tendinous
OS SESAMOIDES	Ses2 aponeurosis	tendinous
OS SESAMOIDES	HFI D1	tendinous
OS SESAMOIDES	HFI D2	tendinous
OS SESAMOIDES	HFI D3	tendinous
TIBFIB	Ses1 aponeurosis	tendinous
tibiale	Ses1 aponeurosis	tendinous
fibulare	Ses1 aponeurosis	tendinous
TIBFIB	Ses2 aponeurosis	tendinous
tibiale	Ses2 aponeurosis	tendinous
fibulare	Ses2 aponeurosis	tendinous
Ses1 aponeurosis	Ses2 aponeurosis	tendinous
Ses1 aponeurosis	HFII D1	tendinous
Ses1 aponeurosis	HFII D2	tendinous
Ses1 aponeurosis	HFIII D3	tendinous
Ses2 aponeurosis	HFI D1	tendinous
Ses2 aponeurosis	HFII D2	tendinous
Ses2 aponeurosis	HFIII D3	tendinous
TIBFIB	Ses flexor digitorum	tendo-muscle
tibiale	Ses flexor digitorum	tendo-muscle
fibulare	Ses flexor digitorum	tendo-muscle
Ses flexor digitorum	HFIII D3	tendon flexor
Ses flexor digitorum	HFIV D4	tendon flexor
Ses flexor digitorum	HFIII D5	tendon flexor
cranium	fascia dorsalis	muscle
cranium	V2	muscle
cranium	V3	muscle
V2	V3	muscle

V3	V4	muscle
V4	V5	muscle
V5	V6	muscle
V6	V7	muscle
V8	sacral vertebra	muscle
cranium	suprascapula	muscle
fascia dorsalis	humerus	muscle
V4	suprascapula	muscle
cranium	urostyle	muscle
V3	urostyle	muscle
V4	urostyle	muscle
V5	urostyle	muscle
V6	urostyle	muscle
V8	urostyle	muscle
sacral vertebra	urostyle	muscle
V4	ilium	muscle
V5	ilium	muscle
V6	ilium	muscle
V7	ilium	muscle
urostyle	ilium	muscle
pubis	xiphisternum	muscle
pubis	mesosternum	muscle
fascia dorsalis	ilium	muscle
fascia dorsalis	suprascapula	muscle
fascia dorsalis	xiphisternum	muscle
ilium	pubis	muscle
fascia dorsalis	xiphisternum	muscle
fascia dorsalis	mesosternum	muscle
urostyle	femur	muscle
ilium	femur	muscle
ilium	Patella	muscle

ilium	femur	muscle
ilium	Patella	muscle
ilium	Patella	muscle
ilium	femur	muscle
ilium	pubis	muscle
ilium	femur	muscle
ilium	pubis	muscle
ischium	femur	muscle
ischium	femur	muscle
ischium	femur	muscle
ischium	femur	muscle
ischium	femur	muscle
ischium	femur	muscle
ilium	Patella	muscle
ischium	Patella	muscle
ischium	Patella	muscle
ischium	Graciella	tendo-muscle
Graciella	Patella	tendo-muscle
Graciella	TIBFIB	tendo-muscle
ilium	TIBFIB	muscle
ischium	TIBFIB	muscle
femur	tibiale	muscle
femur	fibulare	muscle
femur	tibiale	muscle
femur	fibulare	muscle
femur	TIBFIB	muscle
TIBFIB	tibiale	muscle
femur	Ses1 aponeurosis	tendo-muscle
femur	Ses2 aponeurosis	tendo-muscle
Ses1 aponeurosis	PREH PROX	tendinous
Ses2 aponeurosis	PREH PROX	tendinous

PREH DIST	PREH PROX	tendinous
TIBFIB	HFII D1	tendinous
femur	HFII D2	tendinous
TIBFIB	HFII D2	tendinous
femur	HFIII D3	tendinous
TIBFIB	HFIII D3	tendinous
TIBFIB	tibiale	tendo-muscle
TIBFIB	fibulare	tendo-muscle
tibiale	fibulare	tendo-muscle
TIBFIB	PREH PROX	tendo-muscle
TIBFIB	HFIII D3	tendo-muscle
TIBFIB	HFIII D4	tendo-muscle
TIBFIB	HFIII D5	tendo-muscle
tibiale	EL Y(H)	muscle
fibulare	EL Y(H)	muscle
TIBFIB	tibiale	muscle
TIBFIB	METT 2	muscle
TIBFIB	METT 3	muscle
TIBFIB	METT 4	muscle
fibulare	METT 5	muscle
femur	PREH PROX	tendo-muscle
femur	PREH DIST	tendo-muscle
TIBFIB	PREH PROX	tendo-muscle
TIBFIB	PREH DIST	tendo-muscle
femur	METT 1	tendo-muscle
femur	METT 1	tendo-muscle
femur	HFI D1	tendo-muscle
femur	METT 2	tendo-muscle
femur	HFI D2	tendo-muscle
femur	METT 3	tendo-muscle
femur	HFI D3	tendo-muscle

femur	METT 4	tendo-muscle
femur	HFI D4	tendo-muscle
femur	HFI D5	tendo-muscle
femur	HFI D5	tendo-muscle
femur	HFII D3	tendo-muscle
TIBFIB	METT 1	tendo-muscle
TIBFIB	METT 1	tendo-muscle
TIBFIB	HFI D1	tendo-muscle
TIBFIB	METT 2	tendo-muscle
TIBFIB	HFI D2	tendo-muscle
TIBFIB	METT 3	tendo-muscle
TIBFIB	HFI D3	tendo-muscle
TIBFIB	METT 4	tendo-muscle
TIBFIB	HFI D4	tendo-muscle
TIBFIB	HFI D5	tendo-muscle
TIBFIB	HFI D5	tendo-muscle
TIBFIB	HFII D3	tendo-muscle
METT 4	HFI D4	muscle
METT 4	HFII D4	muscle
METT 4	HFIII D4	muscle
METT 4	HFI D5	muscle
METT 4	HFII D5	muscle
TARSAL 2-3	METT 1	muscle
TARSAL 2-3	HFI D1	muscle
TARSAL 2-3	METT 1	muscle
fibulare	METT 2	muscle
fibulare	METT 1	muscle
fibulare	METT 3	muscle
fibulare	METT 4	muscle
METT 2	HFI D2	muscle
METT 3	HFI D3	muscle

METT 4	HFI D4	muscle
METT 5	HFI D5	muscle
METT 1	METT 2	muscle
METT 2	METT 3	muscle
METT 3	METT 4	muscle
METT 4	METT 5	muscle
HFI D3	HFII D3	muscle
HFI D4	HFII D4	muscle
HFI D5	HFII D5	muscle
METT 4	METT 5	muscle
fibulare	METT 5	muscle
fibulare	METT 1	muscle
fibulare	PREH PROX	muscle
fibulare	PREH DIST	muscle
fibulare	HFI D1	muscle
fibulare	HFI D2	muscle
fibulare	HFI D3	muscle
fibulare	HFI D4	muscle
fibulare	HFII D4	muscle
fibulare	HFI D5	muscle
tibiale	HFI D1	muscle
fibulare	HFI D1	muscle
tibiale	HFI D2	muscle
fibulare	HFI D2	muscle
tibiale	HFI D3	muscle
fibulare	HFI D3	muscle
EL Y(H)	HFII D1	muscle
METT 1	HFII D1	muscle
METT 1	HFI D2	muscle
METT 2	HFII D2	muscle
METT 2	HFIII D3	muscle

METT 3	HFIII D3	muscle
tibiale	METT 4	muscle
tibiale	HFI D4	muscle
tibiale	HFIV D4	muscle
METT 4	HFIV D4	muscle
METT 5	HFIV D4	muscle
METT 5	HFIII D5	muscle
PREH PROX	METT 1	muscle
EL Y(H)	METT 1	muscle
cranium	suprascapula	muscle
cranium	scapula	muscle
cranium	scapula	muscle
V3	scapula	tendon
V4	scapula	muscle
V3	scapula	muscle
V3	scapula	muscle
V4	scapula	muscle
suprascapula	scapula	tendo-muscle
coracoid	humerus	muscle
suprascapula	humerus	tendo-muscle
mesosternum	humerus	muscle
epicoracoid	humerus	muscle
mesosternum	humerus	muscle
xiphisternum	humerus	muscle
coracoid	humerus	muscle
episternum	radioulna	tendo-muscle
omosternum	radioulna	tendo-muscle
coracoid	radioulna	tendo-muscle
clavicle	humerus	muscle
procoracoid	humerus	muscle
scapula	humerus	muscle

omosternum	humerus	muscle
scapula	radioulna	tendo-muscle
humerus	radioulna	tendo-muscle
humerus	EL Y(F)	tendo-muscle
humerus	radiale	tendo-muscle
humerus	palmar sesamoid	tendo-muscle
radioulna	palmar sesamoid	muscle
humerus	radioulna	tendo-muscle
humerus	CARPAL 3-4-5	tendo-muscle
humerus	radioulna	tendo-muscle
humerus	METC 3	tendo-muscle
humerus	METC 4	tendo-muscle
humerus	METC 5	tendo-muscle
radioulna	ulnare	muscle
humerus	pararadial	muscle
pararadial	radiale	muscle
humerus	CARPAL 3-4-5	muscle
humerus	ulnare	muscle
humerus	radioulna	muscle
radioulna	PREP PROX	muscle
radioulna	PREP DIST	muscle
ulnare	PREP PROX	muscle
ulnare	PREP DIST	muscle
CARPAL 3-4-5	PREP PROX	muscle
CARPAL 3-4-5	PREP DIST	muscle
CARPAL 3-4-5	FFII D2	tendo-muscle
CARPAL 3-4-5	FFI D2	tendo-muscle
CARPAL 3-4-5	glide metc D2	tendo-muscle
glide metc D2	FFI D2	tendo-muscle
CARPAL 3-4-5	METC 2	muscle
CARPAL 3-4-5	FFII D3	muscle

palmar sesamoid	FFII D3	tendon
CARPAL 3-4-5	glide metc D3	tendo-muscle
glide metc D3	FFI D3	tendo-muscle
METC 3	FFI D3	tendo-muscle
CARPAL 3-4-5	METC 3	tendo-muscle
palmar sesamoid	FFIII D4	tendon
palmar sesamoid	FFII D4	muscle
palmar sesamoid	glide metc D4	tendo-muscle
glide metc D4	FFI D4	tendo-muscle
FFI D4	glide inter FFII-I D4	tendo-muscle
glide inter FFII-I D4	FFII D4	tendo-muscle
METC 4	FFI D4	muscle
CARPAL 3-4-5	METC 4	tendo-muscle
palmar sesamoid	FFIII D5	tendon
palmar sesamoid	FFII D5	tendo-muscle
CARPAL 3-4-5	glide metc D5	tendo-muscle
glide metc D5	FFI D5	tendo-muscle
palmar sesamoid	glide metc D5	tendo-muscle
glide metc D5	FFI D5	tendo-muscle
CARPAL 3-4-5	METC 5	muscle
CARPAL 3-4-5	METC 5	muscle
CARPAL 3-4-5	FFI D5	muscle
palmar sesamoid	METC 5	muscle
CARPAL 3-4-5	METC 5	muscle
radioulna	METC 5	muscle
ulnare	METC 5	muscle
METC 5	FFI D5	muscle
FFI D5	glide inter FFII-I D5	tendo-muscle
glide inter FFII-I D5	FFII D5	tendo-muscle
METC 2	METC 3	muscle
METC 3	METC 4	muscle

METC 4	METC 5	muscle
ulnare	CARPAL 3-4-5	muscle
humerus	METC 2	tendo-muscle
radioulna	METC 2	tendo-muscle
radioulna	FFII D2	tendo-muscle
ulnare	FFII D2	tendo-muscle
EL Y(F)	METC 2	tendo-muscle
CARPAL 2	METC 2	tendo-muscle
METC 2	FFII D2	tendo-muscle
EL Y(F)	METC 2	muscle
CARPAL 2	METC 2	muscle
ulnare	FFI D3	tendo-muscle
EL Y(F)	FFII D3	tendo-muscle
CARPAL 2	FFII D3	tendo-muscle
radiale	FFII D3	tendo-muscle
METC 3	FFII D3	tendo-muscle
ulnare	FFI D4	tendo-muscle
CARPAL 3-4-5	FFI D4	tendo-muscle
EL Y(F)	FFII D4	tendo-muscle
CARPAL 2	FFII D4	tendo-muscle
radiale	FFII D4	tendo-muscle
METC 4	FFIII D4	tendo-muscle
METC 3	FFIII D4	tendo-muscle
METC 5	FFIII D4	tendo-muscle
CARPAL 3-4-5	FFI D5	tendo-muscle
METC 5	FFIII D5	tendo-muscle
METC 4	FFIII D5	tendon
CARPAL 3-4-5	METC 5	muscle
