# Supplementary material for: Delimiting the boundaries of sesamoid identities under the network theory framework
Source: PeerJ. 2020 Aug 17;8:e9691. doi: 10.7717/peerj.9691 (PMC7439958; doi:10.7717/peerj.9691)
Supplement: Supplemental Information 4 — S, sesamoid; CSE: canonical skeletal element, Cat: Skeletal categories compared, S: Significance, GS: glide sesamoid, ES: embedded sesamoid, K-W: Kruskal Wallis Test, W: Wilcoxon pairwise comparison, * statically significance, ** highly statically significance, –non statically significance. [file peerj-08-9691-s004.docx]

Supplemental Table S2: Summary of Kruskal Wallis tests. S, sesamoid; CSE: canonical skeletal element, Cat: Skeletal categories compared, S: Significance, GS: glide sesamoid, ES: embedded sesamoid, K-W: Kruskal Wallis Test, W: Wilcoxon pairwise comparison, * statically significance, ** highly statically significance, -- non statically significance.

| Node parameter | Cat. | Test | Mean compared | | | p-value | S |
| --- | --- | --- | --- | --- | --- | --- | --- |
| Degree | SE-SG-CSE | K-W | 6.10 | 2.07 | 7.32 | 5.81E-08 |  |
|  | SE-CSE | W | 6.10 | 7.32 |  | 0.54 | **--** |
|  | SG-CSE | W | 2.07 | 7.32 |  | 2.00E-08 | ****** |
|  | SE-SG | W | 6.10 | 2.07 |  | 3.00E-04 | ****** |
| Closeness |  |  |  |  |  |  |  |
|  | SE-SG-CSE | K-W | 0.27 | 0.22 | 2.72E-01 | 2.46E-08 | *** |
|  | SE-CSE | W | 0.27 | 2.72E-01 |  | 0.86 | -- |
|  | SG-CSE | W | 0.22 | 2.72E-01 |  | 1.80E-08 | *** |
|  | SE-SG | W | 0.27 | 0.27 |  | 5.30E-05 | *** |
| Betweenness | SE-SG-CSE | K-W | 80.86 | 0.86 | 195.97 | 4.41E-06 | *** |
|  | SE-CSE | W | 80.86 | 195.97 |  | 0.14 | -- |
|  | SG-CSE | W | 0.86 | 195.97 |  | 2.90E-06 | *** |
|  | SE-SG | W | 80.86 | 0.86 |  | 6.40E-02 | -- |
| Eigen-centrality | SE-SG-CSE | K-W | 0.14 | 0.02 | 0.14 | 0.04 | * |
|  | SE-CSE | W | 0.14 | 0.14 |  |  | -- |
|  | SG-CSE | W | 0.02 | 0.14 |  |  | -- |
|  | SE-SG | W | 0.14 | 0.02 |  |  | * |
