# Supplementary material for: Delimiting the boundaries of sesamoid identities under the network theory framework
Source: PeerJ. 2020 Aug 17;8:e9691. doi: 10.7717/peerj.9691 (PMC7439958; doi:10.7717/peerj.9691)
Supplement: Supplemental Information 5 — Centrality values of network elements. Abbreviations: ID: Node identification number; Cat.: Node skeletal category; D.: degree; B.: betweenness; C: closeness: E-C: eigen-centrality; ES: embedded sesamoid; GS: glide sesamoid; CSE: canonical skeletal element, F: finger FP: forelimb phalanx, HF: hindlimb phalanx, (f) forelimb, (h): hindlimb, T: toes, V: vertebra. [file peerj-08-9691-s005.docx]

**Supplemental Table S3.** Centrality values of network elements.

**Legend**: Centrality values of network elements. Abbreviations: ID: Node identification number; Cat.: Node skeletal category; D.: degree; B.: betweenness; C:  closeness: E-C: eigen-centrality; ES: embedded sesamoid; GS: glide sesamoid; CSE: canonical skeletal element, F: finger FP: forelimb phalanx, HF: hindlimb phalanx, (f) forelimb, (h): hindlimb, T: toes, V: vertebra.

| **Skeletal element** | **ID** | **Cat.** | **D.** | **C.** | **B.** | **E-C** |
| --- | --- | --- | --- | --- | --- | --- |
| cranium | 1 | CSE | 7.000 | 0.319 | 334.264 | 0.010 |
| atlas | 2 | CSE | 2.000 | 0.243 | 0.000 | 0.001 |
| V2 | 3 | CSE | 3.000 | 0.244 | 0.000 | 0.002 |
| V3 | 4 | CSE | 5.000 | 0.315 | 0.000 | 0.013 |
| V4 | 5 | CSE | 6.000 | 0.328 | 216.847 | 0.038 |
| V5 | 6 | CSE | 4.000 | 0.289 | 0.000 | 0.039 |
| V6 | 7 | CSE | 4.000 | 0.282 | 0.000 | 0.039 |
| V7 | 8 | CSE | 2.000 | 0.277 | 0.000 | 0.029 |
| V8 | 9 | CSE | 2.000 | 0.249 | 0.000 | 0.010 |
| sacral vertebra | 10 | CSE | 4.000 | 0.282 | 0.500 | 0.039 |
| urostyle | 11 | CSE | 9.000 | 0.328 | 600.588 | 0.097 |
| ilium | 12 | CSE | 13.000 | 0.380 | 2102.166 | 0.412 |
| sacral sesamoid | 13 | SE | 2.000 | 0.277 | 0.000 | 0.027 |
| ischium | 14 | CSE | 6.000 | 0.321 | 248.126 | 0.471 |
| pubis | 15 | CSE | 4.000 | 0.316 | 175.484 | 0.128 |
| suprascapula | 16 | CSE | 6.000 | 0.310 | 241.418 | 0.006 |
| cleithrum | 17 | CSE | 2.000 | 0.242 | 0.000 | 0.001 |
| scapula | 18 | CSE | 8.000 | 0.316 | 112.599 | 0.010 |
| clavicle | 19 | CSE | 6.000 | 0.266 | 10.809 | 0.002 |
| coracoid | 20 | CSE | 5.000 | 0.263 | 37.504 | 0.003 |
| episternum | 21 | CSE | 4.000 | 0.226 | 7.661 | 0.000 |
| omosternum | 22 | CSE | 3.000 | 0.260 | 20.641 | 0.001 |
| procoracoid | 23 | CSE | 3.000 | 0.258 | 9.698 | 0.001 |
| epicoracoid | 24 | CSE | 6.000 | 0.262 | 77.624 | 0.001 |
| mesosternum | 25 | CSE | 6.000 | 0.306 | 167.737 | 0.011 |
| xiphisternum | 26 | CSE | 4.000 | 0.303 | 75.931 | 0.012 |
| humerus | 27 | CSE | 21.000 | 0.342 | 2171.441 | 0.008 |
| radioulna | 28 | CSE | 13.000 | 0.284 | 177.687 | 0.004 |
| radiale | 29 | CSE | 10.000 | 0.274 | 238.838 | 0.001 |
| ulnare | 30 | CSE | 10.000 | 0.276 | 320.989 | 0.002 |
| EL Y(F) | 31 | CSE | 6.000 | 0.267 | 90.405 | 0.001 |
| PREP PROX | 32 | CSE | 5.000 | 0.232 | 10.007 | 0.001 |
| PREP DIST | 33 | CSE | 4.000 | 0.231 | 5.472 | 0.001 |
| CARPAL 2 | 34 | CSE | 4.000 | 0.222 | 0.617 | 0.000 |
| CARPAL 3-4-5 | 35 | CSE | 17.000 | 0.282 | 210.455 | 0.003 |
| METC 2 | 36 | CSE | 9.000 | 0.273 | 194.987 | 0.001 |
| METC 3 | 37 | CSE | 7.000 | 0.272 | 42.047 | 0.001 |
| METC 4 | 38 | CSE | 7.000 | 0.273 | 18.495 | 0.001 |
| METC 5 | 39 | CSE | 9.000 | 0.275 | 120.106 | 0.002 |
| FFI D2 | 40 | CSE | 4.000 | 0.224 | 20.166 | 0.000 |
| FFII D2 | 41 | CSE | 5.000 | 0.232 | 12.756 | 0.001 |
| FFI D3 | 42 | CSE | 4.000 | 0.223 | 22.174 | 0.000 |
| FFII D3 | 43 | CSE | 7.000 | 0.230 | 35.195 | 0.000 |
| FFI D4 | 44 | CSE | 6.000 | 0.229 | 42.699 | 0.000 |
| FFII D4 | 45 | CSE | 7.000 | 0.226 | 87.357 | 0.000 |
| FFIII D4 | 46 | CSE | 5.000 | 0.225 | 8.541 | 0.000 |
| FFI D5 | 47 | CSE | 5.000 | 0.226 | 8.700 | 0.001 |
| FFII D5 | 48 | CSE | 4.000 | 0.221 | 135.229 | 0.000 |
| FFIII D5 | 49 | CSE | 4.000 | 0.222 | 2.150 | 0.000 |
| HFII D1 | 50 | CSE | 5.000 | 0.270 | 77.777 | 0.141 |
| HFI D1 | 51 | CSE | 11.000 | 0.281 | 201.935 | 0.338 |
| METT 1 | 52 | CSE | 11.000 | 0.281 | 6.451 | 0.521 |
| HFII D2 | 53 | CSE | 6.000 | 0.274 | 18.420 | 0.201 |
| HFI D2 | 54 | CSE | 10.000 | 0.281 | 117.076 | 0.360 |
| METT 2 | 55 | CSE | 11.000 | 0.282 | 67.879 | 0.424 |
| HFIII D3 | 56 | CSE | 8.000 | 0.278 | 14.688 | 0.284 |
| HFII D3 | 57 | CSE | 5.000 | 0.273 | 50.579 | 0.177 |
| HFI D3 | 58 | CSE | 10.000 | 0.281 | 146.817 | 0.337 |
| METT 3 | 59 | CSE | 9.000 | 0.283 | 29.779 | 0.399 |
| HFIV D4 | 60 | CSE | 5.000 | 0.234 | 26.210 | 0.094 |
| HFIII D4 | 61 | CSE | 5.000 | 0.272 | 177.037 | 0.100 |
| HFII D4 | 62 | CSE | 6.000 | 0.235 | 29.483 | 0.114 |
| HFI D4 | 63 | CSE | 8.000 | 0.281 | 182.977 | 0.296 |
| METT 4 | 64 | CSE | 13.000 | 0.288 | 143.679 | 0.452 |
| HFIII D5 | 65 | CSE | 4.000 | 0.270 | 190.466 | 0.090 |
| HFII D5 | 66 | CSE | 4.000 | 0.227 | 40.053 | 0.076 |
| HFI D5 | 67 | CSE | 8.000 | 0.281 | 127.712 | 0.352 |
| METT 5 | 68 | CSE | 6.000 | 0.236 | 33.302 | 0.267 |
| PREH DIST | 69 | CSE | 4.000 | 0.274 | 10.450 | 0.205 |
| PREH PROX | 70 | CSE | 8.000 | 0.278 | 14.556 | 0.357 |
| EL Y(H) | 71 | CSE | 6.000 | 0.234 | 9.751 | 0.212 |
| TARSAL 1 | 72 | CSE | 4.000 | 0.231 | 3.400 | 0.087 |
| TARSAL 2-3 | 73 | CSE | 7.000 | 0.238 | 14.200 | 0.216 |
| tibiale | 74 | CSE | 15.000 | 0.289 | 111.406 | 0.601 |
| fibulare | 75 | CSE | 21.000 | 0.297 | 147.102 | 0.732 |
| TIBFIB | 76 | CSE | 28.000 | 0.358 | 2110.552 | 1.000 |
| femur | 77 | CSE | 23.000 | 0.357 | 581.758 | 0.991 |
| glide mett D1 | 78 | SG | 2.000 | 0.223 | 0.000 | 0.051 |
| glide mett D2 | 79 | SG | 2.000 | 0.223 | 0.309 | 0.047 |
| glide inter HFII-I D3 | 80 | SG | 2.000 | 0.221 | 0.467 | 0.031 |
| glide mett D3 | 81 | SG | 2.000 | 0.224 | 0.610 | 0.044 |
| glide inter HFIII-II D4 | 82 | SG | 2.000 | 0.217 | 0.000 | 0.013 |
| glide inter HFII-I D4 | 83 | SG | 2.000 | 0.221 | 0.583 | 0.024 |
| glide mett D4 | 84 | SG | 2.000 | 0.225 | 0.450 | 0.045 |
| glide inter HFII-I D5 | 85 | SG | 2.000 | 0.221 | 0.726 | 0.025 |
| glide mett D5 | 86 | SG | 2.000 | 0.221 | 1.444 | 0.037 |
| CARTILAGO SESAMOIDE | 87 | SE | 6.000 | 0.272 | 4.183 | 0.157 |
| Ses1 aponeurosis | 88 | SE | 11.000 | 0.281 | 61.837 | 0.297 |
| Ses2 aponeurosis | 89 | SE | 11.000 | 0.281 | 56.528 | 0.308 |
| OS SESAMOIDES | 90 | SE | 7.000 | 0.277 | 4.804 | 0.216 |
| Ses flexor digitorum | 91 | SE | 6.000 | 0.273 | 68.815 | 0.167 |
| fascia dorsalis | 92 | CSE | 6.000 | 0.366 | 1988.170 | 0.028 |
| Patella | 93 | SE | 3.000 | 0.278 | 0.000 | 0.160 |
| Graciella | 94 | SE | 3.000 | 0.271 | 76.943 | 0.097 |
| palmar sesamoid | 95 | SE | 10.000 | 0.275 | 535.513 | 0.001 |
| pararadial | 96 | SE | 2.000 | 0.259 | 0.000 | 0.001 |
| glide metc D2 | 97 | SG | 2.000 | 0.221 | 0.000 | 0.000 |
| glide metc D3 | 98 | SG | 2.000 | 0.221 | 2.042 | 0.000 |
| glide metc D4 | 99 | SG | 2.000 | 0.221 | 1.930 | 0.000 |
| glide inter FFII-I D4 | 100 | SG | 2.000 | 0.189 | 0.000 | 0.000 |
| glide metc D5 | 101 | SG | 3.000 | 0.226 | 4.404 | 0.000 |
| glide inter FFII-I D5 | 102 | SG | 2.000 | 0.186 | 0.000 | 0.000 |
